# Robot-Assisted Radical Prostatectomy Performed with the Novel Hugo™ RAS System: A Systematic Review and Pooled Analysis of Surgical, Oncological, and Functional Outcomes

**DOI:** 10.3390/jcm13092551

**Published:** 2024-04-26

**Authors:** Filippo Marino, Stefano Moretto, Francesco Rossi, Carlo Gandi, Filippo Gavi, Riccardo Bientinesi, Marco Campetella, Pierluigi Russo, Francesco Pio Bizzarri, Eros Scarciglia, Mauro Ragonese, Nazario Foschi, Angelo Totaro, Nicolò Lentini, Roberta Pastorino, Emilio Sacco

**Affiliations:** 1 Department of Urology, Fondazione Policlinico Universitario Agostino Gemelli IRCCS, 00168 Rome, Italy; dottorfrancescorossi@gmail.com (F.R.); carlo.gandi@guest.policlinicogemelli.it (C.G.); filippo.gavi01@icatt.it (F.G.); riccardo.bientinesi@policlinicogemelli.it (R.B.); pierluigi92.russo@gmail.com (P.R.); francescopiobizzarri1994@gmail.com (F.P.B.); erosscarciglia@gmail.com (E.S.); mauro.ragonese@policlinicogemelli.it (M.R.); nazario.foschi@policlinicogemelli.it (N.F.); dr.atotaro@gmail.com (A.T.); 2 Department of Medicine and Translational Surgery, Università Cattolica Del Sacro Cuore, 00168 Rome, Italy; marco.campetella@yahoo.it (M.C.); emilio.sacco@unicatt.it (E.S.); 3 Department of Urology, Addenbrooke’s Hospital, Cambridge University Hospitals NHS Foundation Trust, Cambridge CB2 0QQ, UK; 4 Department of Urology, Humanitas Clinical and Research Center, 20089 Milan, Italy; stefano.moretto@humanitas.it; 5 Department of Biomedical Sciences, Humanitas University, 20090 Milan, Italy; 6 Department of Urology, Ospedale Isola Tiberina—Gemelli Isola, 00186 Rome, Italy; 7 Department of Life Sciences and Public Health, Section of Hygiene, Università Cattolica del Sacro Cuore, 00168 Rome, Italy; nicolo.lentini@unicatt.it (N.L.); roberta.pastorino@unicatt.it (R.P.); 8 Department of Woman and Child Health and Public Health—Public Health Area, Fondazione Policlinico Universitario Agostino Gemelli IRCCS, 00168 Rome, Italy

**Keywords:** robot-assisted radical prostatectomy, Hugo RAS system, robotic surgery, outcomes, prostate cancer

## Abstract

**Background/Objectives**: to assess surgical, oncological, and functional outcomes of robot-assisted radical prostatectomy (RARP) performed using the novel Hugo™ RAS system. **Methods**: A systematic review was conducted following the PRISMA guidelines, using PubMed, Web of Science, Scopus, and Embase databases. Eligible papers included studies involving adult males undergoing RARP with the Hugo™ RAS platform, with at least ten patients analyzed. The pooled analysis was performed using a random-effect model. **Results**: Quantitative analysis was conducted on 12 studies including 579 patients. The pooled median docking time, console time, and operative time were 11 min (95% CI 7.95–14.50; I^2^ = 98.4%, ten studies), 142 min (95% CI 119.74–164.68; I^2^ = 96.5%, seven studies), and 176 min (95% CI 148.33–203.76; I^2^ = 96.3%, seven studies), respectively. The pooled median estimated blood loss was 223 mL (95% CI 166.75–280.17; I^2^ = 96.5%, eleven studies). The pooled median length of hospital stay and time to catheter removal were 2.8 days (95% CI 1.67–3.89; I^2^ = 100%, ten studies) and 8.3 days (95% CI 5.53–11.09; I^2^ = 100%, eight studies), respectively. The pooled rate of postoperative CD ≥ 2 complications was 4.1% (95% CI 1–8.5; I^2^ = 63.6%, eleven studies). The pooled rate of positive surgical margins and undetectable postoperative PSA were 20% (95% CI 12.6–28.5; I^2^ = 71.5%, nine studies) and 94.2% (95% CI 87.7–98.6; I^2^ = 48.9%, three studies), respectively. At three months, a pooled rate of social continence of 81.9% (95% CI 73.8–88.9; I^2^ = 66.7%, seven studies) was found. Erectile function at six months was 31% in one study. **Conclusions**: despite the preliminary nature of the evidence, this systematic review and pooled analysis underscores the feasibility, safety, and reproducibility of the Hugo™ RAS system in the context of RARP.

## 1. Introduction

Since the Food and Drug Administration (FDA) approved the first robotic platform for surgical procedures in 2000, robotic-assisted surgery has witnessed the worldwide adoption of the daVinci robotic systems (Intuitive Surgical Inc., Sunnyvale, CA, USA), with their well-established efficacy and safety [1,2]. With the expiration of Intuitive’s patent in 2019, a surge of new robotic systems entered the global market, aiming to enhance surgical capabilities and address the cost-efficiency challenges in healthcare systems. The introduction of the Hugo™ robotic-assisted surgery (RAS) system (Medtronic, Minneapolis, MN, USA), which was first approved for clinical use in urologic procedures by the Panama healthcare regulatory agency in 2021, marked a significant milestone in this evolution [3].

The Hugo™ RAS system, characterized by its portability, configuration flexibility, open console, and some advanced features, offers a design that facilitates the customization of surgical approaches, which may lead to surgical outcomes at least similar to those achieved via established robotic platforms while simultaneously improving cost-effectiveness [4]. 

About three years after its introduction, there is a dearth of comprehensive data analyzing its performance and clinical outcomes, particularly in robot-assisted radical prostatectomy (RARP) procedures, which have been pivotal in the evolution of minimally invasive surgery [5].

This systematic review and pooled analysis aim to synthesize available clinical data on RARP performed using the Hugo™ RAS system. By doing so, we sought to assess whether the surgical outcomes and benefits, historically attributed to the daVinci systems, can be matched, or even surpassed by the Hugo™ RAS system, thereby contributing to a more cost-effective and accessible surgical landscape.

## 2. Material and Methods

### 2.1. Search Strategy

We registered the study protocol in the International Prospective Register of Ongoing Systematic Reviews (PROSPERO registration ID: CRD42023460042) on 13th September 2023. The systematic review was conducted according to the principles highlighted by the European Association of Urology (EAU) Guidelines Office [6], the updated Preferred Reporting Items for Systematic Reviews and Meta-Analyses (PRISMA) recommendation [7], and published guidance on performing meta-analysis on prevalence data [8]. The review question was defined according to the PICOS framework (Supplementary Table S1) [9]. A systematic literature search was conducted in January 2024, with weekly updates until publication, using the PubMed/Medline, Web of Science, Scopus, and Embase databases. No language restrictions were applied. The references of significant studies were then manually analyzed to identify studies of interest. A detailed overview of the search strategy is available in Supplementary S1.

### 2.2. Study Selection and Eligibility Criteria

Studies were deemed eligible for the analysis if they (1) included at least ten adult males (aged ≥ 18 years) diagnosed with prostate cancer, (2) evaluated the performance of the Hugo™ RAS System in performing transperitoneal RARP, and (3) assessed surgical and/or oncological and/or functional outcomes. Both prospective and retrospective studies, reported in full-text or as conference abstracts, were included. A comparator was not required. We excluded reviews with or without meta-analysis, commentaries, authors’ replies, theses, and case reports.

Initial screening was performed independently by two investigators (F.M. and S.M.) based on the titles and abstracts of the articles to identify ineligible reports. Potentially relevant studies were subjected to a full-text review, and the relevance of the reports was confirmed after the data extraction process. Duplicated studies from the same author’s group were excluded, retaining the ones fulfilling the selection criteria and the most recent ones; however, if they fulfilled the selection criteria and provided additional information on outcomes of interest, they were retained for these outcomes only. The authors of the eligible studies were contacted twice via email in case of missing or dubious data. Disagreements were resolved via consultation with a third co-author (E.S.). 

### 2.3. Data Extraction and Outcome Measures

Data extraction was performed by two independent reviewers (F.M. and S.M.) with disagreements resolved via discussion until a consensus was reached. A data extraction form was used to extract equivalent information in a standardized manner. To minimize the intra-examiner variability, all the extracted data were double-checked.

Surgical outcomes were docking time, console time, overall operative time, nerve-sparing surgery rate, lymphadenectomy rate, number of lymph nodes removed, intra- and postoperative complications rate [Clavien–Dindo (CD) scale ≥ 2], estimated blood loss (EBL), length of hospital stay (LoS), and time to catheter removal. Oncological outcomes were the positive surgical margin (PSM) rate and postoperative prostate specific antigen (PSA) value.

Functional outcomes were time to urinary continence recovery, postoperative continence status, and erectile function. For the functional outcomes, we used the following definitions: social continence as the use of no more than one pad per day [10], incontinence cure as no pad use, and erectile function as the ability to achieve an erection. 

In addition to the outcomes of interest, the following variables were extracted in a Microsoft Excel spreadsheet: name of the first author, year of publication, country, study design, sample size, average age, body mass index (BMI), preoperative PSA level, prostate volume, clinical T stage, and International Society of Urological Pathology (ISUP) grade group at biopsy.

### 2.4. Study Quality Assessment

Two authors (F.M. and S.M.) independently evaluated the study quality. The risk of bias (RoB) was assessed according to EAU guidelines for systematic case series reviews [6]. The overall RoB level was judged considering five closed-ended questions: the presence of a pre-established protocol, the inclusion of the entire population or consecutive participant selection, the completeness of outcome data and explanation of any missing data, the reporting of all predetermined outcomes, and the proper measurement of outcomes. Answers can be “Yes”, “No”, or “Unclear”. If the answer to all five questions is “Yes”, the study is at “low” RoB. If the answer to any question is “No” or “Unclear”, the study is at “high” RoB. RoB assessment results were displayed using the robvis tool [11]. Disagreements were resolved via consultation with a third co-author (E.S.).

### 2.5. Statistical Analysis

We undertook an initial descriptive analysis of the studies, followed by a pooled analysis evaluating the surgical, oncological, and functional outcomes. A pooled analysis also summarized baseline patient characteristics.

Pooled estimates were obtained using the median and interquartile range for continuous variables; for this analysis, the Quantile Matching Estimation (QEmedian) method was adopted [12], which uses the standard inverse variance to pool study-specific medians. For dichotomous variables, pooled estimates were obtained using the inverse variance method, with event rates obtained through the Freeman-Tukey double-arcsine transformation method to stabilize the variance of each study’s proportion [13]. Meta-analysis was performed using a random-effect model [8]. The DerSimonian-Laird estimator [14] was used to estimate the between-study variance in dichotomous variables, while the restricted maximum-likelihood (REML) estimator was employed in continuous variables [15]. All pooled estimates were reported as percentages or medians with a 95% confidence interval (CI). The results were graphically presented in forest plots. 

Heterogeneity among the outcomes of included studies was evaluated using Cochrane’s Q-test and the I^2^ statistic. Subgroup and meta-regression analyses were carried out to investigate sources of heterogeneity and evaluate effect modification exclusively for outcomes supported by a minimum of ten studies [16]. Possible confounders were defined by the consensus of two authors from a literature review. Subgroup analysis was performed based on the RoB, whereas meta-regression analysis considered continuous covariates such as sample size, age, and study design. 

A leave-one-out sensitivity analysis was performed to explore the influence of a single study on the pooled effect estimate by iteratively removing one study at a time to ensure pooled estimates and statistical heterogeneity were not driven by a single study [17]. 

Statistical analyses were performed using OpenMetaAnalyst software and R version 4.3.2 (31 October 2023) for Windows. Statistical significance was set at a *p*-value < 0.05.

## 3. Results

### 3.1. Study Selection and Characteristics

**Figure 1:**
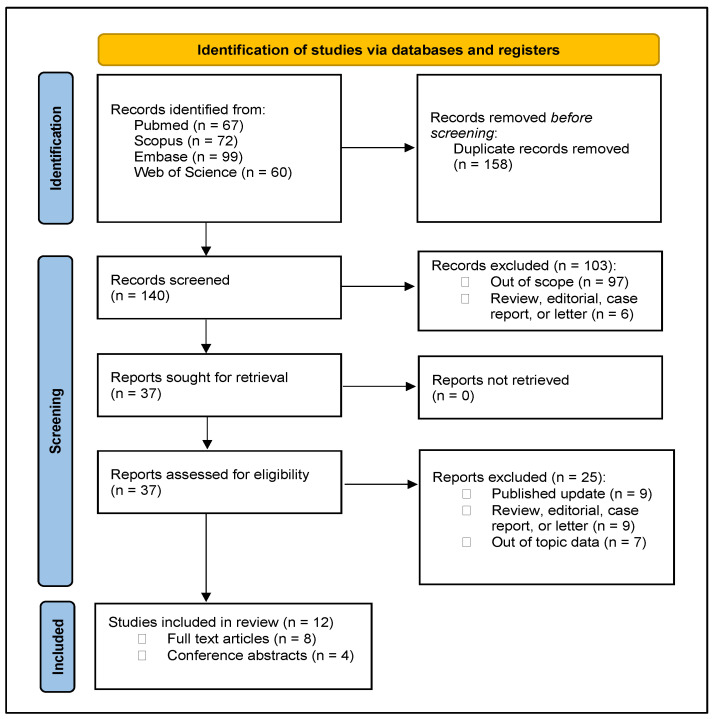
PRISMA flow diagram for the selection process.

We summarized the study selection process in the PRISMA flowchart (Figure 1). 

The literature search retrieved 298 records. We assessed 37 documents, and 12 papers met our inclusion criteria, including 579 patients. Four studies were conference abstracts [18,19,20,21], and eight were reported in full-text [3,22,23,24,25,26,27,28]. Five citations [29,30,31,32,33] were duplicated studies by the same group of authors and were retained exclusively because they provided additional data compared to the most recent included report [22,23,26,28]. Specifically, Bravi et al. [29] provide additional data on postoperative PSA and console time, while Totaro et al. [30], Ragavan et al. [31], Ou et al. [32], and Veccia/Antonelli et al. [33] provide additional information on docking parameters.

The included studies were published between February 2023 and April 2024. Six studies used a comparative design [18,23,24,25,26,28], one of which was a randomized study [18]. For our analysis, only data from the arm of patients undergoing RARP with the Hugo™ RAS system were extracted from these studies. Eight studies were retrospective [3,19,20,21,23,24,26,27], and only four [18,22,25,28] used a prospective design. Supplementary Table S2 provides a complete description of the reasons for exclusion after a full-text review. The authors of three studies provided the required additional data [18,20,22]. 

### 3.2. Risk of Bias

**Figure 2:**
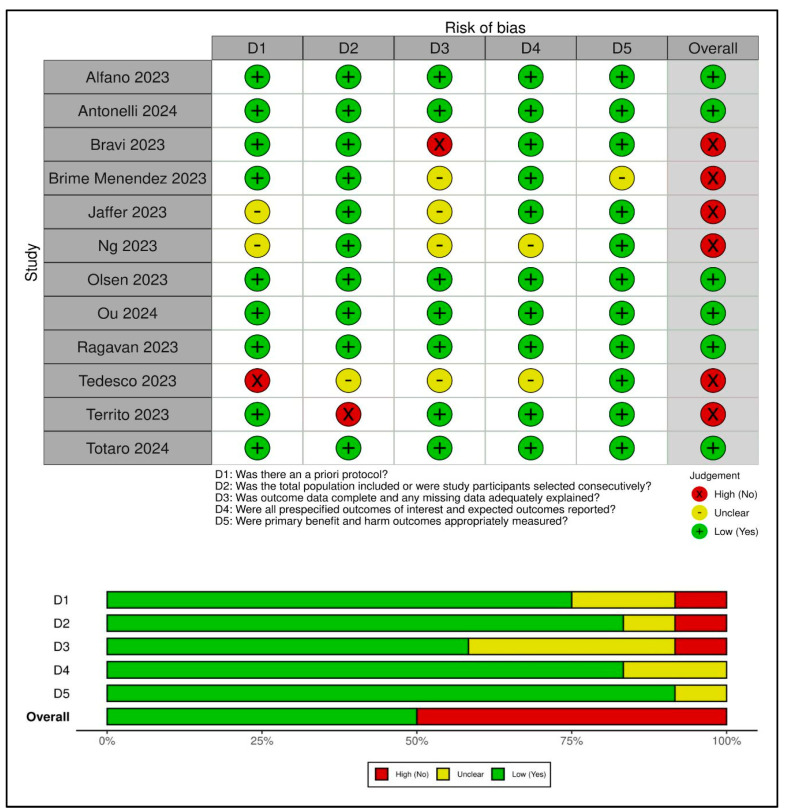
Risk of bias assessment [3,18,19,20,21,22,23,24,25,26,27,28].

We summarized RoB assessments in Figure 2. Six studies were judged to be at low RoB [3,22,24,25,26,28] and six at high RoB [18,19,20,21,23,27]. The most frequent biases were incomplete outcome data and inadequate explanations for missing data (five studies) [18,19,20,21,23] and the absence of a priori protocol (three studies) [19,20,21].

### 3.3. Baseline Patient Characteristics

Baseline patient characteristics are displayed in Table 1. The pooled median patients’ age and BMI were 65.7 years (95% CI 64.7–66.7; I^2^ = 46.3%) and 25.9 (95% CI 25.6–26.2; I^2^ = 0%), respectively. The preoperative PSA value was reported for 518 patients from ten studies [3,18,20,21,22,23,25,26,27,28] with a pooled median value of 7.55 ng/mL (95% CI 6.88–8.21; I^2^ = 57%). The pooled median prostate volume of 506 patients from nine studies [3,18,20,22,23,24,26,27,28] was 42.8 cc (95% CI 40.5–45; I^2^ = 31.8%). At the preoperative prostate biopsy histology, 32.8% of patients (152 out of 466 from six studies [3,18,22,23,26,28]) had an ISUP grade group ≥ 3 (more detailed data in Supplementary Table S3). The rate of palpable disease was 38.3% (176 out of 459 patients from eight studies [3,18,22,23,24,25,26,27]), and the suspicion of extracapsular extension at magnetic resonance imaging was reported in 36 out of 181 patients (21.4%) from two studies [23,27].

### 3.4. Surgical Outcomes

The surgical outcomes are displayed in Table 2. All procedures included were performed using a transperitoneal approach. Specific surgical techniques were reported from five studies: standard anterograde transperitoneal technique [25,28], modified apical dissection and lateral prostatic fascia preservation technique [3], Montsouris technique [22], and Aalst technique [23]. Patients were placed either in a supine position [22,23,27,28] or in a head-down Trendelenburg position [22,23,25,26,27,28]. The lithotomy position was also adopted to allow the placement of the endoscope cart between the patient’s legs [3,22,25]; in two studies, the endoscope cart was positioned on the left side [23,26]. Antonelli et al. [28,33] implemented a standardized setting with the endoscope cart positioned on the left side and an alternative “mirrored” setting with the endoscope cart positioned on the right side. The adopted docking and tilt angles, reported in eight studies [3,19,23,25,26,27,28], are summarized in Supplementary Table S4. 

**Figure 3:**
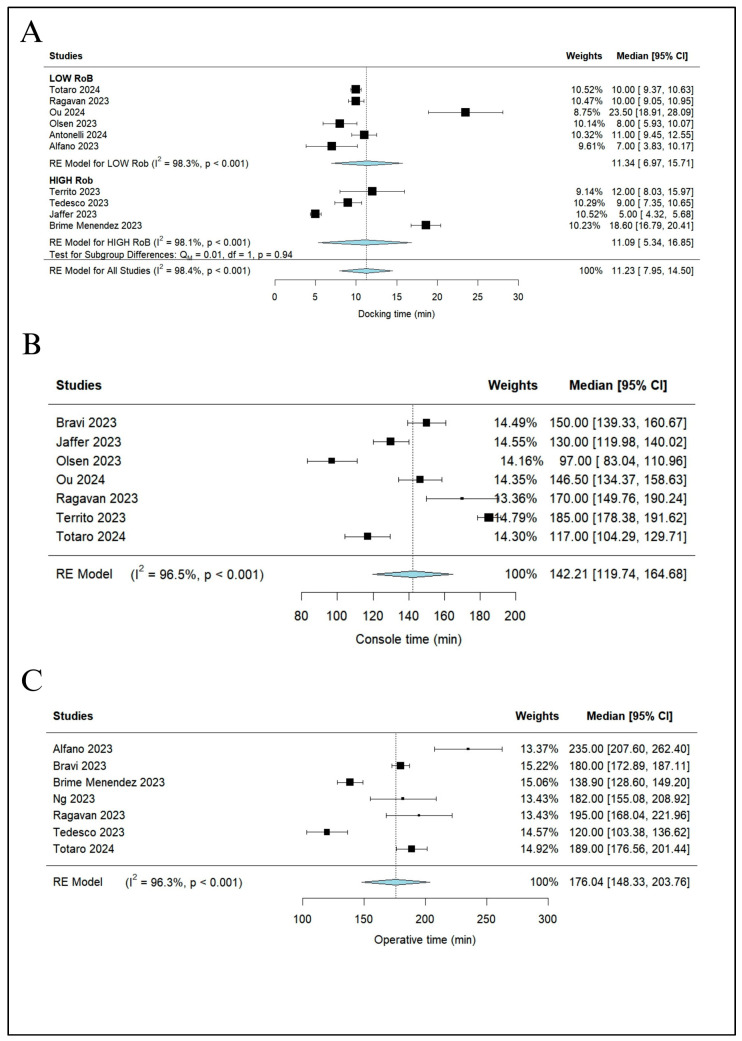
Duration of surgery: (**A**) docking time with subgroup analysis based on risk of bias, (**B**) console time, and (**C**) operative time. CI, confidence interval [3,19,20,21,22,23,24,25,26,27,28].

The duration of surgery was reported in ten studies and divided into docking time, console time, and operative time. The pooled median docking time of 357 procedures from ten studies [3,18,19,21,22,24,25,26,27,28] was 11.23 min (95% CI 7.95–14.50; I^2^ = 98.4%) (Figure 3A), the pooled median console time of 347 procedures from seven studies [18,21,23,24,25,26,27] was 142.21 min (95% CI 119.74–164.68; I^2^ = 96.5%) (Figure 3B), and the pooled median operative time of 443 procedures from seven studies [3,18,19,20,22,23,25] was 176.04 min (95% CI 148.33–203.76; I^2^ = 96.3%) (Figure 3C).

**Figure 4:**
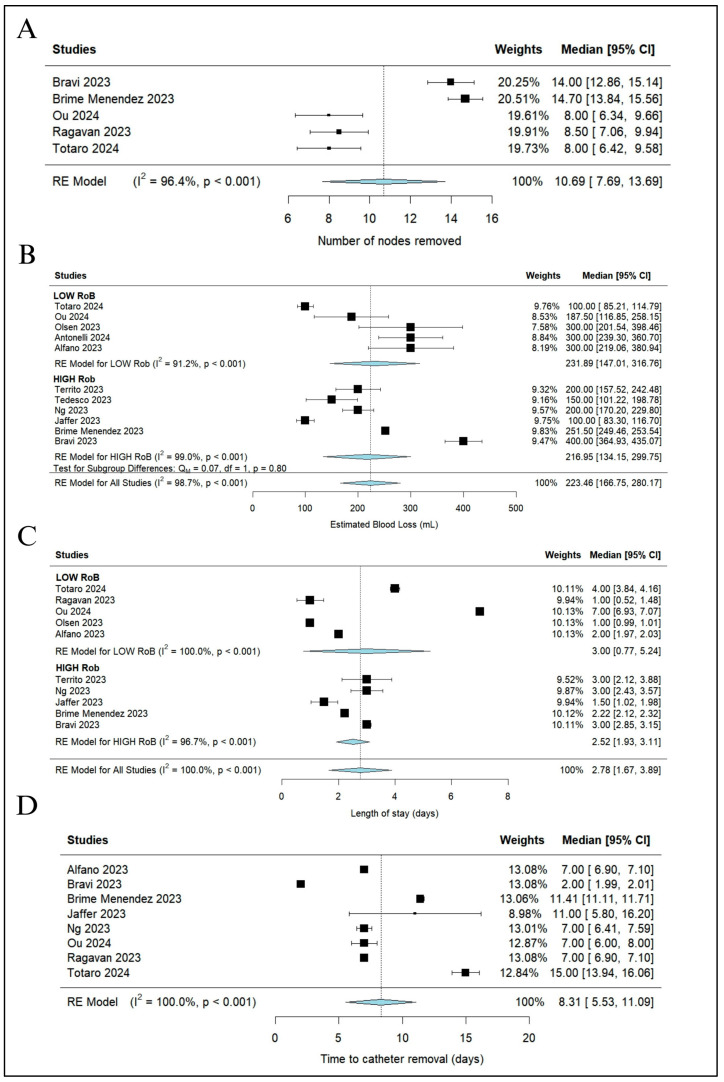
Continuous variables: (**A**) number of nodes removed, (**B**) estimated blood loss with subgroup analysis based on risk of bias, (**C**) length of stay with subgroup analysis based on risk of bias, and (**D**) time to catheter removal. CI, confidence interval [3,19,20,21,22,23,24,25,26,27,28].

A nerve-sparing procedure was performed in 61.9% of patients (252 out of 407 patients from six studies [18,22,23,24,25]) and a pelvic lymphadenectomy in 38% of patients (120 out of 422 patients from six studies [3,18,22,23,24,25]). The pooled median number of nodes removed was 10.69 (95% CI 7.69–13.69; I^2^ = 96.4%) (418 patients from five studies [18,22,23,25,26]) (Figure 4A).

EBL was reported in 11 of 12 studies [3,18,19,20,21,22,23,24,26,27,28] with a pooled median value of 223.46 mL (95% CI 166.75–280.17; I^2^ = 98.7%) (Figure 4B).

The pooled median LoS was 2.78 days (95% CI 1.67–3.89; I^2^ = 100%) (Figure 4C) for 499 patients from ten studies [3,18,20,21,22,23,24,25,26,27] and the pooled median time to catheter removal was 8.31 days (95% CI 5.53–11.09; I^2^ = 100%) (Figure 4D) for 463 patients from eight studies [3,18,20,21,22,23,25,26]. 

**Figure 5:**
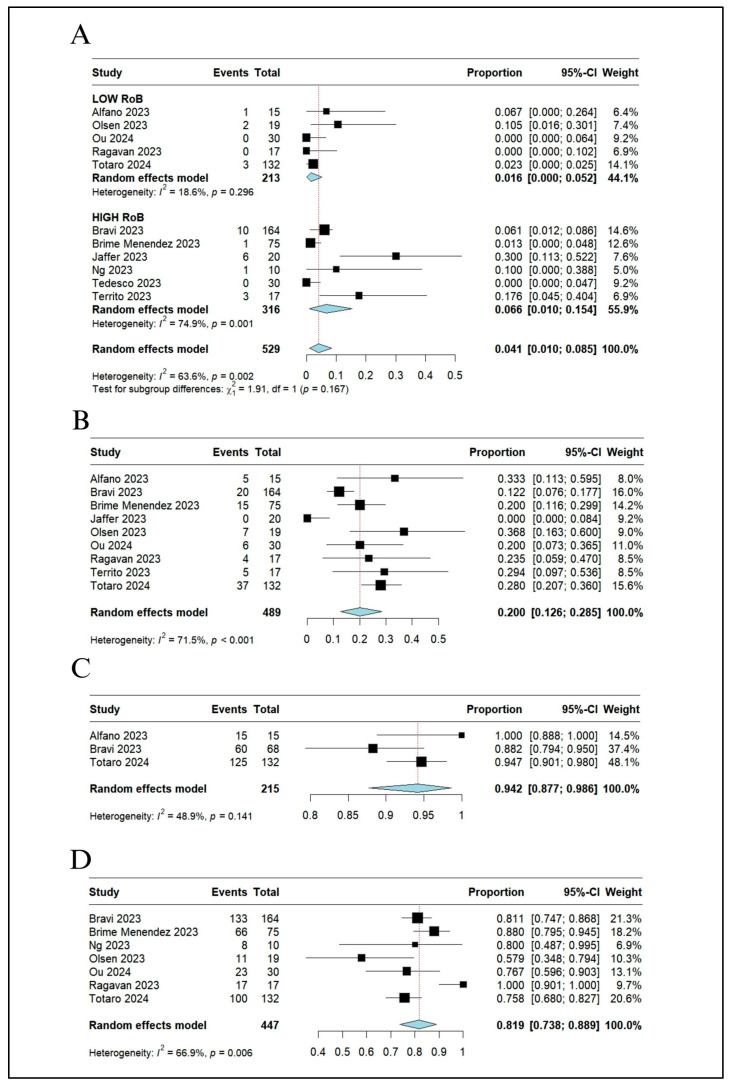
Dichotomous variables: (**A**) Clavien–Dindo complications ≥ 2 with subgroup analysis based on risk of bias, (**B**) positive surgical margins, (**C**) undetectable PSA level at first follow-up after surgery, and (**D**) social continence at 3 months. PSA, prostate specific antigen; CI, confidence interval [3,19,20,21,22,23,24,25,26,27,28].

Eleven studies reported data on postoperative complications [3,18,19,20,21,22,23,24,25,26,27]. CD ≥ 2 complications occurred in 27 out of 529 patients. The pooled rate of postoperative CD ≥ 2 complications was 4.1% (95% CI 1–8.5%; I^2^ = 63.6%) (Figure 5A). Bleeding complications occurred in five patients: gastrointestinal bleeding due to gastritis [3], acute bleeding from the abdominal wall after drainage removal that spontaneously stopped [22], pelvic hematoma [27], pelvic bleeding requiring a trans-arterial embolization [27], and hematuria [20]. Postoperative lymphocele requiring transient positioning of percutaneous drainage was reported in one study [22]. One patient experienced acute urinary retention treated with catheterization [23].

Two CD 3b complications requiring re-surgery were reported: a jejunal perforation during adherence lysis due to a previous gastrojejunostomy [22] and ileus due to a hernia at the port hole treated with diagnostic laparoscopy and intravenous antibiotics administration [24]. Two studies [19,25] reported the absence of CD ≥ 2 complications. Conversion to open surgery was never necessary. 

Antonelli et al. [28] reported three intraoperative complications graded according to the intraoperative adverse incident classification proposed by the EAU ad hoc complications guidelines panel [34]. Specifically, a bladder wall injury and a small bowel superficial injury due to cautery were managed through suturing and repair. Additionally, one instance of catheter entrapment during the suture for anastomosis was resolved by the catheter’s liberation.

At subgroup analysis, RoB did not explain heterogeneity in EBL, LoS, and docking time pooled analysis. RoB partly explained the heterogeneity in CD ≥ 2 complication pooled analysis, with lower heterogeneity in the low-risk group (I^2^ = 18.6%). 

At meta-regression (Supplementary Table S5), only age showed a statistically significant association with CD ≥ 2 complications (R^2^ = 64.8%), with higher age associated with fewer complications. Supplementary Figure S1 shows the corresponding bubble plot.

### 3.5. Oncological Outcomes

The oncological outcomes are displayed in Table 3. Final histopathology results demonstrated a rate of 70% of patients with pT1a-pT2c tumor stage (331 patients from eight studies [3,18,21,22,23,24,26,27]) and 28.8% with pT3a-pT4b (136 patients from eight studies [3,18,21,22,23,24,26,27]). A pathological node stage was reported only in three studies [18,22,23] with a rate of 19% of patients with N0 (70 patients) and 5% with N1 or N2 stage (18 patients) (Supplementary Table S6). The rate of the ISUP 1–2 grade group was 66% (285 patients from seven studies [3,18,21,22,23,26,27]) and the rate of the ISUP 3–5 grade group was 34% (150 patients from seven studies [3,18,21,22,23,26,27]). (Supplementary Table S7).

A total of nine studies [3,18,21,22,23,24,25,26,27] with four hundred eighty-nine patients reported the rate of PSM at final histopathology, ranging from 0% (none of twenty patients [21]) to 37% (seven of nineteen patients [24]). Only one study [21] reported no PSM. The two studies including more than 100 patients reported a PSM rate of 12% (20 of 164 patients [23]) and 28% (37 of 132 patients [22]). The pooled rate of PSM after RARP was 20% (95% CI 12.6–28.5%; I^2^ = 71.5%) (Figure 5B). None of the studies under review provided information regarding the location, number, and length of PSM. 

Seven studies [3,18,22,24,25,27,29] reported postoperative PSA levels. An undetectable PSA level was observed at the first follow-up after surgery (one to three months) in 160 out of 183 patients (85%) from three studies [3,22,29]. The pooled rate of undetectable PSA after surgery was 94.2% (95% CI 87.7–98.6%; I^2^ = 48.9%) (Figure 5C). The pooled median PSA level three months after surgery was 0.07 ng/mL (95% CI 0.00–0.07%) from four studies [18,24,25,27] including 128 patients. 

### 3.6. Functional Outcomes

The functional outcomes are displayed in Table 3. Data on urinary continence status three months after surgery was reported in seven studies [18,20,22,23,24,25,26], including 395 patients. The pooled rate of social continence at three months after surgery was 81.9% (95% CI 73.8–88.9%; I^2^ = 66.9%) (Figure 5D). Only one study [24] reported a cure rate as high as 58% (11 out of 19 patients) at three months.

One study [29] of 112 patients reported that the median time to urinary continence recovery was 36 days (95% CI 34–44%), and the probability of urinary continence recovery was 36% (95% CI 28–47%) at one month and 81% (95% CI 72–89%) at three months.

Erectile function was assessed only in four studies [22,24,25,26] including 198 patients. Erections at three months were reported in five out of nineteen (26.3%) patients [24], in twenty-two out of thirty (73.3) patients [26], and in none out of seventeen patients [25]. Only one study reported a rate of erections at six months [22] that was as high as 31% overall (25 out of 81 patients); 88% of patients receiving a nerve-sparing surgery (60% bilateral and 28% monolateral) were considered to have recovered. 

### 3.7. Sensitivity Analysis

At leave-one-out sensitivity analysis, the pooled estimates remained stable when one study was removed, suggesting that no individual study was substantially influential (Supplementary Table S8). However, in the pooled analysis of undetectable PSA level at the first follow-up after surgery, the study by Bravi et al. [23] turned out to be very influential on heterogeneity (I^2^ = 0.0% after exclusion); in the pooled analysis concerning CD ≥ 2 complications, the study conducted by Jaffer et al. [21] was found to have a slight influence on heterogeneity (I^2^ = 42.8% after exclusion). 

## 4. Discussion

### 4.1. Main Findings and Interpretation of the Results

The results of this systematic review and pooled analysis show that satisfactory surgical, oncological, and functional outcomes can be achieved by performing RARP with the new Hugo™ RAS robotic platform, underscoring the procedure’s feasibility, safety, and efficacy.

In their meta-analysis of RARP performed using the daVinci systems, Novara et al. [35] reported a mean operative time of 152 min, a mean EBL of 166 mL, a mean time to catheter removal of 6.3 days, and a mean hospital stay of 1.9 days. Although it is difficult to perform reliable comparisons, based on the aforementioned meta-analysis, our results suggest slightly lower performance with Hugo™ RAS for these outcomes. These differences may be due to several reasons: the early nature of the included series, implying a possible learning curve, differences in study populations and outcome measures, and specific meta-analysis methodologies. Accordingly, in the largest published study contrasting Hugo™ RAS (164 patients) and da Vinci robotic (378 patients) systems [23], the median operative time was slightly higher in the Hugo™ RAS series (180 min vs 165 min; *p* = 0.05), likely reflecting the learning curve and more complex docking associated with the new platform. 

Regarding post-RARP complications, our pooled estimate appears to be consistent with those reported in recent meta-analyses: Bertolo et al. [36] reported a CD ≥ 2 complication rate of 7.6%, and Wang et al. [1] reported a major complication (CD > 3) rate of 2.5% with an overall complication rate of 13.7%.

Based on oncological outcomes, the system proved to be effective in prostate cancer surgeries. The observed pooled rate of PSM (20%) compares favorably with those found in previous RARP series, which showed rates ranging from 6.5% to 30.3% [37,38,39,40]. The pooled rate of undetectable postoperative PSA was also promising, although the assessment of postoperative oncological outcomes in the current review was constrained due to the limited data provided and the very short follow-up period.

Looking at functional outcomes, the Hugo™ RAS system achieved satisfactory postoperative social urinary continence rates (74% and 81.9% at one and three months postsurgery, respectively). Comparable figures were reported in the RARP series using the more-established robotic platforms. According to a recent review by Wang et al. [1], social continence rates (defined as using 0–1 pad per day) after RARP (fifteen prospective studies and one randomized control trial) were 62.3% (95% CI: 60.5–63.9%) at three months and 87.1% (95% CI: 83.9–89.8%) at six months.

Our study draws insights from various authors who have provided perspectives based on their experiences with the Hugo™ RAS system. The study by Totaro et al. [22] highlighted the initial challenges encountered during the early procedures, with the Hugo™ RAS system reporting yellow and red errors, especially during the first nine procedures. The reported errors were primarily technical issues related to the robotic arms. However, after the ninth procedure and a major software update, the authors observed a significant reduction in the occurrence of these errors. 

Accordingly, Antonelli et al. [28], in their comparative study between Hugo^TM^ RAS and daVinci systems, reported a greater number of malfunction events in the Hugo cases, which significantly affected procedure flow and total procedure time. Additionally, their study identified the breakpoint from learning to proficiency in 22 cases.

Ou et al. [26,32] focused on troubleshooting and pauses during surgery. The authors observed that early cases had more troubleshooting pauses that decreased significantly with increased experience. The pre-console preparation was remarkably more efficient starting from the seventh case. Notably, they suggested surgeons familiar with daVinci systems experienced a smoother transition to the Hugo™ RAS system, minimizing adverse effects. Bravi et al. [23] also suggested that surgical skills can safely be transferred to this new technology.

Alfano et al. [3] and Ragavan et al. [25] reported initial challenges in the docking process, emphasizing the need for training due to the unique configuration of the Hugo™ RAS system. However, once docked, Alfano et al. [3] reported that the system provided appropriate traction and dissection capacity without delaying or interfering with intraoperative performance. The operative time was compatible with what they usually performed using other robotic platforms.

Regarding the console system, Ragavan et al. [25] noted more accessible communication with the surgical team and less strain on the neck while working with the open console of the Hugo™ RAS system compared to the closed console of the daVinci platform.

The comparative study by Olsen et al. [24] explored the daVinci to Hugo™ RAS platform skill transfer. The authors demonstrated that experienced robotic surgeons could switch between systems without a clinically relevant performance dip. They observed a greater mental load when using the Hugo™ RAS system but emphasized the need for multi-platform training to accommodate surgeons switching between different systems.

Jaffer et al. [21] expressed satisfaction with the image quality and maneuverability of the Hugo™ RAS system. The authors found the needle drivers particularly efficient, demonstrating the safe and effective integration of the Hugo™ RAS platform into an established robotic surgery program.

The study by Territo et al. [27] emphasized the importance of ex-vivo training in laboratories to tailor cart positioning and docking settings. The authors recommended studies to investigate the learning curve and outcomes of the Hugo™ RAS system in novice robotic surgeons. They also highlighted the need for a steep learning curve in established robotic centers adopting the novel platform. Accordingly, Ragavan [25] emphasizes the importance of a learning curve and system familiarity.

Overall, the studies suggested a positive outlook for this new platform’s application in RARP procedures; although, the system requires surgeons and medical teams to familiarize themselves with new operational protocols. The authors’ primary impressions concerning the utilization of the Hugo^TM^ RAS system, encompassing both the benefits and drawbacks of its various aspects, are outlined in Supplementary Table S9, along with the main differences compared to the daVinci platform.

### 4.2. Limitations

The present study has some limitations due to the relatively recent introduction of the Hugo™ RAS system and inherent drawbacks of systematic reviews of prevalence data that may be plagued by issues related to differences between the included studies [8]. First of all, the limited number of papers included in the systematic review and the relatively small sample size across the included studies may have impacted the precision of the summary effects. Another potential limitation is the retrospective nature and the high RoB of most of the selected studies, which may have jeopardized the accuracy and reliability of the results. Additionally, the relatively short follow-up data after surgery did not allow the evaluation of more meaningful outcomes, such as 12-month continence status, sexual function, and biochemical recurrence. The absence of possible unpublished data may have introduced a publication bias partly limited by our comprehensive literature search that also included conference abstracts. A formal publication bias analysis was not performed due to the limited number of studies, the high statistical heterogeneity, and because there is a lack of published research or guidance regarding the evaluation of publication bias in meta-analyses of proportional data [8]. 

The included studies varied in sample size, study design, population characteristics, and follow-up durations, translating to high heterogeneity. As a result, our summary estimates should also be considered with caution because, despite conducting subgroup and meta-regression analysis to address this diversity, the heterogeneity remained largely unexplained. Specifically, although promising, findings regarding functional outcomes require careful interpretation due to the limited sample size and short follow-up period. Further research is needed to assess these outcomes over longer follow-up durations to enhance their reliability. Finally, the body of evidence included early case series where a significant learning curve is likely expected that may have affected the results in the direction of worse outcomes [40]. 

### 4.3. Implications for Practice and Future Research

To our knowledge, our present systematic review and pooled analysis is the first comprehensive assessment of the surgical outcomes and early oncological and functional outcomes for RARP performed using the novel Hugo™ RAS system. Our findings can be considered demonstrative of the Hugo™ RAS system’s practicality and reliability in performing RARP, though further scrutiny for long-term outcomes is required. This review’s implications extend to both clinical practice and avenues for future research; although, clinicians should interpret our findings cautiously, considering the early stage of evidence accumulation. In addition to the well-known benefits of robot-assisted surgery, the Hugo™ RAS system appears to offer additional advantages such as enhanced visualization and dexterity; improved ergonomics; increased versatility and adaptability due to the modular design; additional safety features; simulation modules; open consoles improving team communication and allowing multi-tasking possibilities, data collection, and analysis via a cloud-based, AI-powered surgical video capture and a management platform (Medtronic’s Touch Surgery Enterprise); and, last but not least, long-term cost savings [3,22,23,24,26].

Although the available literature suggests that the Hugo™ RAS system holds its ground against other time-honored robotic systems, a more exhaustive assessment of whether this new platform can match or surpass outcomes historically attributed to the daVinci platform carries implications for cost-effectiveness and robotic surgery accessibility. Thus, given the dearth of long-term and comparative data on the Hugo™ RAS system, researchers should aim to fill existing gaps by conducting larger longitudinal and possibly comparative studies with extended follow-up periods. Direct comparisons with established robotic platforms would provide valuable benchmarks for evaluating the Hugo™ RAS system’s performance, although performing randomized trials in the surgical field is notoriously difficult. Future studies should also delve into the potential learning curve associated with the Hugo™ RAS system for expert and naïve console surgeons. A more in-depth evaluation of the outcomes’ transferability from Intuitive platforms to this new system will contribute to a nuanced understanding of the system’s applicability. 

## 5. Conclusions

This systematic review and pooled analysis pioneers a more comprehensive evaluation of the Hugo™ RAS system in the context of RARP, shedding light on its initial outcomes. While recognizing the preliminary nature of the body of evidence and the need for further research, this review underscores the feasibility, safety, and reproducibility of the Hugo™ RAS system in performing the surgical procedure that most benefited from the robotic surgery. The studies collectively suggest that experienced robotic surgeons can successfully transition to the Hugo™ RAS system without compromising meaningful outcomes; although, initial challenges and the importance of a learning curve have been acknowledged by most authors. Continuous technical improvements, system updates, and tailored training programs appear to be crucial for overcoming these challenges and fully realizing the potential of the Hugo™ RAS system in improving surgical outcomes. Further research is required to comprehensively understand the Hugo™ RAS system’s role in robotic-assisted surgery.

## Figures and Tables

**Table 1:** Baseline characteristics.

Author, Year	Country	Type of Study	Number of Patients N	Age (Years) Median (Q1–Q3)	BMI (kg/m^2^) Median (Q1–Q3)	Preoperative PSA Level (ng/mL) Median (Q1–Q3)	Prostate Volume (cc) Median (Q1–Q3)	Clinical T Stage Unpalpable (cT1) N (%)	Clinical T Stage Palpable (cT2–T4) N (%)	ISUP 1–2 at Biopsy N (%)	ISUP 3–5 at Biopsy N (%)
Alfano et al., 2023 [3]	Panama	R	15	62 (59–67)	24.9 (23–28)	7.3 (4.8–8.1)	52 (41–56)	8 (53)	7 (47)	13 (87)	2 (13)
Antonelli et al. 2024 [28]	Italy	P	50	65.9 ± 5.9 mean ± SD	25.4 (24.5–27.8)	7.7 (5.9–11)	40 (29–50) (on 44 patients)	NR	NR	27 (54)	23 (46)
Bravi et al., 2023 [23]	Belgium	R	164	65 (60–70)	26 (24–29)	8 (5.7–11.1)	42 (33–58)	109 (66)	55 (34)	111 (67)	53 (33)
Brime Menendez et al., 2023 [18]	Spain	P (RCT)	75	65.8 ± 8.1 mean ± SD	26.2 ± 4 mean ± SD	6.4 ± 1.9 mean ± SD	41.7 ± 16.3 mean ± SD	51 (68)	24 (32)	49 (65.4)	26 (34.6)
Jaffer et al., 2023 [21]	England	R	20	61 (50–72) median (range min-max)	25.7 (22.1–34) median (range min-max)	7.9 (3.8–43) median (range min-max)	NR	NR	NR	NR	NR
Ng et al., 2023 [20]	Hong Kong	R	10	68 (67–71)	NR	9.4 (7.4–12.3)	43 (34–45)	NR	NR	NR	NR
Olsen et al., 2023 [24]	Denmark	R	19	66 (63–73)	25.5 (23.7–27.5)	NR	47 (30–75)	10 (52.6)	9 (47.4)	NR	NR
Ou et al., 2024 [26]	Taiwan	R	30	66.5 (10) median (IQR)	25.79 (4.26) median (IQR)	8.81 (7.66) median (IQR)	40 (16.5) median (IQR)	0 (0)	30 (100)	23 (76.6)	7 (23.3)
Ragavan et al., 2023 [25]	India	P	17	68 (66–72)	24.6 (22.6–26.6)	12.4 (8.8–27)	NR	0 (0)	17 (100)	NR	NR
Tedesco et al., 2023 [19]	Italy	R	30	NR	NR	NR	NR	NR	NR	NR	NR
Territo et al., 2023 [27]	Spain	R	17	64 (59–69)	27 (24–27)	6.4 (5.1–9.4)	35 (30–56)	10 (58.8)	7 (41.2)	NR	NR
Totaro et al., 2024 [22]	Italy	P	132	66.5 (62–71.5)	26 (24.4–28)	8 (5.5–11.3)	46 (33–62)	105 (79.6)	27 (20.4)	91 (68.9)	41 (31.1)

BMI, Body Mass Index; MRI, Magnetic Resonance Imaging; PSA, Prostatic Specific Antigen; ISUP, International Society of Urological Pathology; NR, Not Reported; SD, Standard Deviation; Q1, First Quartile; Q3, Third Quartile; IQR, Interquartile Range; R, Retrospective; P, Prospective; RCT, Randomized Control Trial.

**Table 2:** Surgical outcomes.

Author, Year	Docking Time (Min) Median (Q1-Q3)	Console Time (min) Median (Q1–Q3)	Operative Time (min) Median (Q1–Q3)	EBL (mL) Median (Q1–Q3)	Nerve-Sparing Surgery (*n*; %)	Lymphadenectomy (*n*; %)	Number of Lymph Nodes Removed Median (Q1–Q3)	LOS (Days) Median (Q1–Q3)	Catheter Removal (Days) Median (Q1–Q3)
Alfano et al., 2023 [3]	7 (15) median (max)	NR	235 (213–271)	300 (100–310)	NR	5 (33)	NR	2 (2–2)	7 (7–7)
Antonelli et al. 2024 [28]	11 (8–14)	NR	NR	300 (150–400)	NR	NR	NR	NR	NR
Bravi et al., 2023 [23]	NR	150 (110–175 on 112 patients)	180 (150–200)	400 (250–500)	147 (90)	41 (25)	14 (10–18)	3 (3–4)	2 (2–2)
Brime Menendez et al., 2023 [18]	18.6 ± 8 mean ± SD	NR	138.9 ± 45.5 mean ± SD	251.5 ± 9 mean ± SD	61 (81.3)	22 (29.3)	14.7 ± 3.8 mean ± SD	2.22 ± 0.42 mean ± SD	11.41 ± 1.34 mean ± SD
Jaffer et al., 2023 [21]	5 (4–8) median (range min-max)	130 (90–150) median (Range min-max)	NR	100 (50–500) median (range min-max)	NR	NR	NR	1.5 (1–4) median (range min-max)	11 (10–43) median (range min-max)
Ng et al., 2023 [20]	NR	NR	182 (171–217)	200 (200–500)	NR	NR	NR	3 (2–3)	7 (7–8)
Olsen et al., 2023 [24]	8 (6–11)	97 (87–120)	NR	300 (150–400)	11 (57.9)	10 (52.6)	NR	1 (1–1)	NR
Ou et al., 2024 [26]	23.5 (22–30.5) (on 12 patients)	146.5 (36.5) median (IQR)	NR	187.5 (242.5) median (IQR)	NR	NR	8 (5) median (IQR)	7 (0) median (IQR)	7 (3) median (IQR)
Ragavan et al., 2023 [25]	10 (5–10) median (range min–max)	170 (160–205)	195 (180–240)	NR	0 (0)	17 (100)	8.5 (6.75–10)	1 (1–2)	7 (7–7)
Tedesco et al., 2023 [19]	9 (7–12)	NR	120 (100–150)	150 (100–250)	NR	NR	NR	NR	NR
Territo et al., 2023 [27]	12 (7–16)	185 (177–192)	NR	200 (150–250)	NR	NR	NR	3 (2–4)	NR
Totaro et al., 2024 [22]	10 (8–12)	117 (96–175)	189 (146–227)	100 (50–150)	33 (25.2)	25 (18.9)	8 (4.5–15)	4 (4–5)	15 (14–20.5)

EBL, Estimated Blood Loss; LOS, Length of Stay; NR, Not Reported; SD, Standard Deviation; Q1, First Quartile; Q3, Third Quartile, IQR, Interquartile Range.

**Table 3:** Oncological and functional outcomes.

Author, Year	Positive Surgical Margins N (%)	Postoperative PSA Levels at 1 Month (ng/mL) Median (Q1–Q3)	Postoperative PSA at 3 Months (ng/mL) Median (Q1–Q3)	Undetectable PSA at 1 Month N (%)	Undetectable PSA at 3 Months N (%)	Social Continence Rate at 1 Month N (%)	Social Continence at 3 Months N (%)	Postoperative Complications (Clavien–Dindo ≥ 2) N (%)	Postoperative Erections N (%) (Follow-Up)
Alfano et al., 2023 [3]	5 (33)	0 (0–0)	NR	15 (100)	NR	9 (61)	NR	1 (6.7)	NR
Antonelli et al., 2024 [28]	NR	NR	NR	NR	NR	NR	NR	NR	NR
Bravi et al., 2023 [23,29]	20 (12)	NR	NR	60 (88%)	NR	108 (66)	133 (81)	10 (6)	NR
Brime Menendez et al., 2023 [18]	15 (20)	0.34 (0.83) median (IQR)	0.07 (0.21) median (IQR)	NR	NR	NR	66 (88)	1 (1.3)	NR
Jaffer et al., 2023 [21]	0 (0)	NR	NR	NR	NR	NR	NR	6 (30)	NR
Ng et al., 2023 [20]	NR	NR	NR	NR	NR	NR	8 (80)	1 (10)	NR
Olsen et al., 2023 [24]	7 (36.8)	NR	0 (0–0.45)	NR	NR	NR	11 (57.9)	2 (10.5)	5 (26.3) (3 months)
Ou et al., 2024 [26]	6 (20)	NR	NR	NR	NR	8 (26.7)	23 (76.6)	0 (0)	22 (73.3) (3 months)
Ragavan et al., 2023 [25]	4 (23.5)	NR	0.07 Median	NR	NR	NR	17 (100)	0 (0)	0 (0) (3 months)
Tedesco et al., 2023 [19]	NR	NR	NR	NR	NR	NR	NR	0 (0)	NR
Territo et al., 2023 [27]	5 (29.4)	NR	0.009 (0.006–0.045)	NR	NR	NR	NR	3 (17.7)	NR
Totaro et al., 2024 [22]	37 (28)	NR	NR	NR	125 (94.6%)	NR	100 (75.7%)	3 (4)	25 (31) (6 months)

PSA, Prostate Specific Antigen; NR, Not Reported; IQR, Interquartile Range; Q1, First Quartile; Q3, Third Quartile.

## Data Availability

The data presented in this study are available on request from the corresponding author.

## Supplementary Materials

The following supporting information can be downloaded at: https://www.mdpi.com/article/10.3390/jcm13092551/s1, S1: Literature search strategy; Table S1: PICOS criteria; Table S2: Exclusion following full-text review; Table S3: Focus on ISUP grade group at the preoperative prostate biopsy; Table S4: Docking arms and patient positioning during RARP; Table S5: Results of the meta-regression analysis; Table S6: Focus on tumor and node stage at the final histopathology; Table S7: Focus on ISUP grade group at the final histopathology; Table S8: Results of the sensitivity analysis; Table S9: Comparison of technical insights between Hugo^TM^ RAS and daVinci systems [41,42,43,44]; Figure S1: Bubble plot of CD ≥ 2 complications.
