# Supplementary material for: Robot-Assisted Radical Prostatectomy Performed with the Novel Hugo™ RAS System: A Systematic Review and Pooled Analysis of Surgical, Oncological, and Functional Outcomes
Source: J Clin Med. 2024 Apr 26;13(9):2551. doi: 10.3390/jcm13092551 (PMC11084580; doi:10.3390/jcm13092551)
Supplement: Supplementary file 1 [file jcm-13-02551-s001.zip › jcm-2945893-supplementary.pdf]

This supplementary material has been provided by the authors to give readers additional information about their work.

List of materials

Supplementary S1. Literature search strategy

Supplementary Table S1. PICOS criteria

Supplementary Table S2. Exclusion following full text review

Supplementary Table S3. Focus on ISUP grade group at the preoperative prostate biopsy

Supplementary Table S4. Docking arms and patient positioning during RARP

Supplementary Table S5. Results of the meta-regression analysis

Supplementary Table S6. Focus on tumor and node stage at the final histopathology

Supplementary Table S7. Focus on ISUP grade group at the final histopathology

Supplementary Table S8. Results of the sensitivity analysis

Supplementary Table S9. Comparison of technical insights between Hugo^{TM} RAS and daVinci systems

Supplementary Figure S1. Bubble plot of length of stay

Supplementary S1. *Literature Search Strategy*

Search strategy - Total results: 297.

Search date: 20th January 2024

Last update: 7th April 2024

PubMed: ("HUGOTM"[All Fields] OR "HUGO"[All Fields] OR "HUGO RAS"[All Fields] OR "HUGO TM"[All Fields]) AND ("robotic-assisted"[All Fields] OR "prostatectomy"[All Fields] OR "radical prostatectomy"[All Fields] OR "robot-assisted radical prostatectomy"[All Fields] OR "robotic surgery"[All Fields] OR "robotic"[All Fields] OR "urologic"[All Fields] OR "RARP"[All Fields]) - Results: 66

Web of Science: (TS=("HUGOTM") OR TS=("HUGO") OR TS=("HUGO RAS") OR TS=("HUGO TM")) AND (TS=("robotic-assisted") OR TS=("prostatectomy") OR TS=("radical prostatectomy") OR TS=("robot-assisted radical prostatectomy") OR TS=("robotic surgery") OR TS=("robotic") OR TS=("urologic") OR TS=("RARP")) - Results: 60

Scopus: (TITLE-ABS-KEY ("HUGOTM") OR TITLE-ABS-KEY ("HUGO") OR TITLE-ABS-KEY ("HUGO RAS") OR TITLE-ABS-KEY ("HUGO TM")) AND (TITLE-ABS-KEY ("robotic-assisted") OR TITLE-ABS-KEY ("prostatectomy") OR TITLE-ABS-KEY ("radical prostatectomy") OR TITLE-ABS-KEY ("robot-assisted radical prostatectomy") OR TITLE-ABS-KEY ("robotic surgery") OR TITLE-ABS-KEY ("robotic") OR TITLE-ABS-KEY ("urologic") OR TITLE-ABS-KEY ("RARP"))- Results: 72

Embase: ('HUGOTM':ab,ti OR 'HUGO':ab,ti OR 'HUGO RAS':ab,ti OR 'HUGO TM':ab,ti) AND ('robotic assisted':ab,ti OR 'prostatectomy'/exp OR 'radical prostatectomy':ab,ti OR 'robotic assisted radical prostatectomy':ab,ti OR 'robotic surgery':ab,ti OR 'robotic':ab,ti OR 'urologic':ab,ti OR 'RARP':ab,ti) - Results: 99

Supplementary Table S1. *PICOS criteria*	
Population	Adult males (aged ≥ 18 years) diagnosed with prostate cancer amenable to local treatment
Intervention	Transperitoneal robot-assisted radical prostatectomy performed with the Hugo™ RAS System
Comparison	A comparator was not required
Outcomes	Surgical, oncological, and functional outcomes
Study design	Randomized controlled trials, prospective and retrospective case series

Supplementary Table S2. *Exclusion following full-text review*		
**Title**	**Author**	**Exclusion criteria**
Robot-assisted Radical Prostatectomy with the Novel Hugo Robotic System: Initial Experience and Optimal Surgical Set-up at a Tertiary Referral Robotic Center	Bravi CA	Published update
Robot-assisted radical prostatectomy feasibility and setting with the Hugo™ robot-assisted surgery system	Sarchi L	RARP on cadavers
HUGO(TM) RAS System in Robotic-Assisted Radical prostatectomy is highligheted in International Brazilian Journal of Urology	Favorito AL	Editorial
Nerve-sparing robot-assisted radical prostatectomy with the HUGO™ robot-assisted surgery system using the 'Aalst technique'	Paciotti M	Published update
Outcomes of Robot-assisted Radical Prostatectomy with the Hugo RAS Surgical System: Initial Experience at a High-volume Robotic Center	Bravi CA [29]	Published update (it has been considered for additional data regarding post-operative PSA and console time)
The new surgical robotic platform HUGOTM RAS: System description and docking settings for robot-assisted radical prostatectomy	Totaro A [30]	Published update (it has been considered for additional data regarding tilt and docking angles)
State of the Art in Robotic Surgery with Hugo RAS System: Feasibility, Safety and Clinical Applications	Prata F	Review
Robotic Surgery in Urology: History from PROBOT® to HUGOTM	Brassetti A	Review
Technology description, initial experience and first impression of HUGO™ RAS robot platform in urologic procedures in Brazil	Carneiro A	No RARP data
Extraperitoneal robot-assisted radical prostatectomy with the Hugo™ RAS system: initial experience of a tertiary center with a high background in extraperitoneal laparoscopy surgery	Marques-Monteiro M	Extraperitoneal RARP
Evaluation of Hugo RAS System in Major Urologic Surgery: Our Initial Experience	Ragavan N [31]	Published update (it has been considered for additional data regarding tilt and docking angles)
Current urological applications of the Hugo™ RAS	Soputro NA	Review

system		
Multimodular robotic systems (Hugo RAS and Versius CMR) for pelvic surgery: tasks and perspectives from the bed-side assistant	Sighinolfi MC	No RARP data
Experiences with and Expectations of Robotic Surgical Systems: A Rapid Qualitative Review [Internet]	Martinello N	Review
New multiport robotic surgical systems: a comprehensive literature review of clinical outcomes in urology	Salkowski M	Review
New robotic platforms for prostate surgery: the future is now	Mjaess G	Review
Comparison of outcomes of multiple platforms for assisted robotic-prostatectomy: rationale and design	Veccia A	Letter to editor
Video comparison of HUGO-RAS vs. daVinci techniques in robotic radical prostatectomy: Providing insights from our preliminary study results	Brime Menendez R	Multimedia abstract
STEP-BY-STEP DESCRIPTION OF ROBOT-ASSISTED RADICAL PROSTATECTOMY WITH THE NOVEL HUGO RAS ROBOTIC SYSTEM USING THE AALST TECHNIQUE	Paciotti M	Abstract with data published in subsequent paper
A SYNOPTIC JOURNEY IN ROBOTIC ASSISTED RADICAL PROSTATECTOMY WITH THREE DIFFERENT PLATFORMS: CLINICAL CASES BY A SINGLE SURGEON WITH THE DA VINCI, HUGO TM RAS AND VERSIUS SURGICAL SYSTEMS	Rocco BM	Abstract with no data available
SURGICAL OUTCOMES OF ROBOT-ASSISTED RADICAL PROSTATECTOMY WITH THE NOVEL HUGO ROBOTIC SYSTEM: INITIAL 100 CASES PERFORMED AT A TERTIARY REFERRAL ROBOTIC CENTER CENTER	Paciotti M	Abstract with data published in subsequent paper
Retzius-sparing radical prostatectomy performed with HugoTM RAS surgical system: Initial experience from a referral center	Dell'Oglio P	Case report
Implementation of HUGO Ras system in robotic-assisted radical prostatectomy	Alfano CG	Abstract with data published in subsequent study
Outcomes of Robotic-Assisted Radical Prostatectomy with Hugo RAS System: Initial Experience in Japan	Tbata K	Undivided data involving intraperitoneal and extraperitoneal RARP

Surgical setting and perioperative outcomes of robot-assisted radical prostatectomy with the novel "hugo" robotic system: initial experience at a tertiary referral robotic center	Paciotti M	Abstract with data published in subsequent study
Robot-assisted radical prostatectomy using hugo RAS system: the pioneer experience in Taiwan and Northeast Asia	Ou YC [32]	Published update (it has been considered for additional data regarding tilt and docking angles)
Mirrored port placement for robotic radical prostatectomy with the Hugo RAS™ System: initial experience	Veccia A/Antonelli A [33]	Published update (it has been considered for additional data regarding tilt and docking angles)

Supplementary Table S3. *Focus on ISUP grade group at the preoperative prostate biopsy.*

**Author, Year**	**Country**	**Number of patients N**	**ISUP 1 at biopsy N (%)**	**ISUP 2 at biopsy N (%)**	**ISUP 3 at biopsy N (%)**	**ISUP 4 at biopsy N (%)**	**ISUP 5 at biopsy N (%)**
Totaro et al., 2024 [22]	Italy	132	43 (32.6)	48 (36.4)	19 (14.4)	14 (10.5)	8 (6.1)
Alfano et al., 2023 [3]	Panama	15	7 (47)	6 (40)	0 (0)	2 (13)	0 (0)
Ragavan et al., 2023 [25]	India	17	NR	NR	NR	NR	NR
Ou et al., 2024 [26]	Taiwan	30	17 (56.7)	6 (20)	3 (10)	3 (10)	1 (3.3)
Bravi et al., 2023 [23]	Belgium	164	53 (32)	58 (35)	41 (25)	10 (6)	2 (2)
Olsen et al., 2023 [24]	Denmark	19	NR	NR	NR	NR	NR
Territo et al., 2023 [27]	Spain	17	NR	NR	NR	NR	NR

Tedesco et al., 2023 [19]	Italy	30	NR	NR	NR	NR	NR
Ng et al., 2023 [20]	Hong Kong	10	NR	NR	NR	NR	NR
Jaffer et al., 2023 [21]	England	20	NR	NR	NR	NR	NR
Brime Menendez et al., 2024 [18]	Spain	75	23 (30.8)	26 (34.7)	19 (25.3)	7 (9.2)	0 (0)
Antonelli et al., 2024 [28]	Italy	50	9 (18)	18 (36)	14 (28)	9 (18)	0 (0)

ISUP, International Society of Urological Pathology; NR, Not Reported.

Supplementary Table S4. Docking arms and patient positioning during RARP

Author, Year	Country	Patient position	Endoscope cart position	Endoscope: Tilt angle – Docking angle (° / °)	Right arm: Tilt angle – Docking angle (° / °)	Left arm: Tilt angle – Docking angle (° / °)	Fourth arm: Tilt angle – Docking angle (° / °)
Totaro et al., 2024 [22, 30]	Italy	Lithotomy<br>0° supine<br>Trendelenburg	Between legs	-45 / 175	-40 / 225	-40 / 140	30 / 105
Alfano et al., 2023 [3]	Panama	Lithotomy	Between legs	-45 / 185	-30 / 230	-30 / 140	15 / 105
Ragavan et al., 2023 [25, 31]	India	Low lithotomy<br>Less than 30°<br>Trendelenburg	Betweem legs	-45 / 185	-30 / 230	-30 /140	15 / 255

Ou et al., 2023 [26,32]	Taiwan	25° Head-down Trendelenburg	Left side	-45 / 175	-30 / 220	-30 / 140	15 / 105
Bravi et al., 2023 [23]	Belgium	Lithotomy 0° supine Trendelenburg	Left side	-45 / 175	-30 / 220	-30 / 140	30 / 105
Olsen et al., 2023 [24]	Denmark	NR	NR	NR	NR	NR	NR
Territo et al., 2023 [27]	Spain	30° Trendelenburg 30° legs abducted Supine	NR	-45 / 175	-30 / 225	-30 / 140	30 / 105
Tedesco et al., 2023 [19]	Italy	NR	NR	-45 / 175	-40 / 225	-40 / 140	*3 cart arm used
Ng et al., 2023 [20]	Hong Kong	NR	NR	NR	NR	NR	NR
Jaffer et al., 2023 [21]	England	NR	NR	NR	NR	NR	NR
Brime Menendez et al., 2024 [22]	Spain	NR	NR	NR	NR	NR	NR
Veccia /Antonelli et al., 2024 [28,33] (Standard setting: bed assistant on the right)	Italy	Lithotomy Legs joined and extended 25° Trendelenburg	Left side	-45 / 175 ± 5	- 45 / 220 ± 5	-30 / 140 ± 5	30 /105 ± 5
Veccia /Antonelli et al., 2024 [28,33] (Mirrored setting: bed assistant on the left)			Right side	-45 / 185 ± 5	- 30 / 220 ± 5	-30 / 140 ± 5	30 /255 ± 5

NR, Not Reported.

Supplementary Table S5. Results of meta-regression analysis

Estimated blood of loss (ml) [3, 18, 21-24, 26, 27]			
Variables	Beta	95% CI	P-value
Age	4.908	-30.393, 40.209	0.785
Sample size	0.634	-0.817, 2.084	0.392
Study design			
Prospective	ref		
Retrospective	60.229	-108.457, 228.915	0.484
R^2 for the model	0.00%		
Length of stay (days) [3, 19, 21-27]			
Variables	Beta	95% CI	P-value
Age	0.282	-0.326, 0.890	0.363
Sample size	0.010	-0.015, 0.034	0.440
Study design			
Prospective	ref		
Retrospective	1.453	-0.788, 4.525	0.354
R^2 for the model	0.0%		
Clavien-Dindo ≥2 complications [3, 18, 21-25, 27]			

Variables	OR	95% CI	P-value
Age	0.959	0.920, 0.999	**0.045**
Sample size	1.000	0.998, 1.001	0.435
Study design			
Prospective	ref		
Retrospective	1.085	0.934, 1.262	0.287
R^2 for the model	64.78%		

Supplementary Table S6. *Focus on tumor and node stage at the final histopathology.*										
**Author, Year**	**Country**	**Number of patients N**	**T1 stage at pathology N (%)**	**T2 stage at pathology N (%)**	**T3 stage at pathology N (%)**	**T4 stage at pathology N (%)**	**Nx stage at pathology N (%)**	**N0 stage at pathology N (%)**	**N1 stage at pathology N (%)**	**N2 stage at pathology N (%)**
Totaro et al., 2024 [22]	Italy	132	2 (1.5)	94 (72.3)	34 (26.2)	0 (0)	107 (81.1)	20 (15.5)	5 (3.8)	0 (0)
Alfano et al., 2023 [3]	Panama	15	0 (0)	11 (73.3)	4 (26.7)	0 (0)	NR	NR	NR	NR
Ragavan et al., 2023 [25]	India	17	NR	NR	NR	NR	NR	NR	NR	NR
Ou et al., 2024 [26]	Taiwan	30	0 (0)	24 (80)	3 (10)	0 (0)	NR	NR	NR	NR
Bravi et al., 2023 [23]	Belgium	164	0 (0)	110 (67)	54 (33)	0 (0)	123 (74.7)	34 (21)	7 (4.3)	0 (0)
Olsen et al., 2023 [24]	Denmark	19	0 (0)	11 (57.9)	8 (42.1)	0 (0)	NR	NR	NR	NR
Territo et al., 2023 [27]	Spain	17	0 (0)	14 (82.4)	3 (17.6)	0 (0)	NR	NR	NR	NR
Tedesco et al., 2023 [19]	Italy	30	NR	NR	NR	NR	NR	NR	NR	NR
Ng et al., 2023 [20]	Hong Kong	10	NR	NR	NR	NR	NR	NR	NR	NR
Jaffer et al., 2023 [21]	England	20	0 (0)	10 (50)	10 (50)	0 (0)	NR	NR	NR	NR
Brime Menendez et al., 2024 [18]	Spain	75	0 (0)	55 (73.3)	20 (26.7)	0 (0)	53 (70.7)	16 (21.3)	6 (8)	0 (0)

NR, Not Reported.

Supplementary Table S7. *Focus on ISUP grade group at the final histopathology.*							
**Author, Year**	**Country**	**Number of patients N**	**ISUP 1 at pathology N (%)**	**ISUP 2 at pathology N (%)**	**ISUP 3 at pathology N (%)**	**ISUP 4 at pathology N (%)**	**ISUP 5 at pathology N (%)**
Totaro et al., 2024 [22]	Italy	132	20 (15.5)	76 (58.9)	30 (23.3)	1 (0.8)	2 (1.5)
Alfano et al., 2023 [3]	Panama	15	2 (13.3)	11 (73.3)	1 (6.7)	0 (0)	1 (6.7)
Ragavan et al., 2023 [25]	India	17	NR	NR	NR	NR	NR
Ou et al., 2023 [32]	Taiwan	12	0 (0)	3 (25)	5 (42)	0 (0)	4 (33)
Bravi et al., 2023 [23]	Belgium	164	19 (12)	83 (51)	48 (29)	10 (6)	4 (2)
Olsen et al., 2023 [24]	Denmark	19	NR	NR	NR	NR	NR
Territo et al., 2023 [27]	Spain	17	1 (5.9)	9 (52.9)	5 (29.4)	0 (0)	2 (11.8)
Tedesco et al., 2023 [19]	Italy	30	NR	NR	NR	NR	NR
Ng et al., 2023 [20]	Hong Kong	10	NR	NR	NR	NR	NR
Jaffer et al., 2023 [21]	England	20	0 (0)	10 (50)	10 (50)	0 (0)	0 (0)
Brime Menendez et al., 2024 [18]	Spain	75	30 (40)	21 (28)	19 (25.3)	4 (5.3)	1 (1.4)

ISUP, International Society of Urological Pathology; NR, Not Reported.

Supplementary Table S8. *Results of the sensitivity analysis.*					
Docking time (min)					
Studies	Effect size	95% CI	P-value	Heterogeneity	Het. P-value
Alfano et al., 2023 [3]	11.69	8.15, 15.22	<0.001	98.6%	<0.001
Antonelli et.al., 2024 [28]	11.28	7.58, 14.98	<0.001	98.7%	<0.001
Brime Menendez et al., 2023 [18]	10.34	7.25, 13.42	<0.001	98.1%	<0.001
Jaffer et al., 2023 [21]	11.95	8.63, 15.26	<0.001	97.7%	<0.001
Olsen et al., 2023 [24]	11.61	8.00, 15.21	<0.001	98.7%	<0.001
Ou et al., 2024 [26]	10.04	7.50, 12.58	<0.001	97.4%	<0.001
Ragavan et al., 2023 [25]	11.39	7.70, 15.08	<0.001	98.4%	<0.001
Tedesco et al., 2023 [19]	11.50	7.85, 15.16	<0.001	98.7%	<0.001
Territo et al., 2023 [27]	11.17	7.52, 14.82	<0.001	98.8%	<0.001
Totaro et al., 2024 [22]	11.39	7.70, 15.09	<0.001	98.1%	<0.001
All studies	11.23	7.95, 14.50	<0.001	98.4%	<0.001
Console time (min)					
Studies	Effect size	95% CI	P-value	Heterogeneity	Het. P-value
Bravi et al., 2023 [23]	140.89	114.43, 167.35	<0.001	97.0%	<0.001

Jaffer et al., 2023 [21]	144.28	118.07, 170.49	<0.001	96.8%	<0.001
Olsen et al., 2023 [24]	149.65	129.43, 169.87	<0.001	95.2%	<0.001
Ou et al., 2024 [26]	141.49	114.95, 168.04	<0.001	97.1%	<0.001
Ragavan et al., 2023 [25]	137.92	113.53, 162.31	<0.001	96.9%	<0.001
Territo et al., 2023 [27]	134.67	114.46, 154.87	<0.001	93.5%	<0.001
Totaro et al., 2024 [22]	146.42	121.69, 171.15	<0.001	96.7%	<0.001
All studies	142.21	119.74, 164.68	<0.001	96.5%	<0.001
Operative time (min)					
Studies	Effect size	95% CI	P-value	Heterogeneity	Het. P-value
Alfano et al., 2023 [3]	166.72	142.19, 191.25	<0.001	95.1%	<0.001
Bravi et al., 2023 [23]	175.56	142.46, 208.66	<0.001	95.5%	<0.001
Brime Menendez et al., 2023 [18]	182.60	153.10, 212.09	<0.001	95.2%	<0.001
Ng et al., 2023 [20]	175.30	142.82, 207.79	<0.001	97.2%	<0.001
Ragavan et al., 2023 [25]	173.22	141.46, 204.99	<0.001	97.1%	<0.001
Tedesco et al., 2023 [19]	185.10	160.51, 209.69	<0.001	94.5%	<0.001
Totaro et al., 2024 [22]	173.98	141.39, 206.57	<0.001	96.5%	<0.001
All studies	176.04	148.33, 203.76	<0.001	96.3%	<0.001

Number of nodes removed					
Studies	Effect size	95% CI	P-value	Heterogeneity	Het. P-value
Bravi et al., 2023 [23]	9.85	6.58, 13.12	<0.001	95.8%	<0.001
Brime Menendez et al., 2023 [18]	9.66	6.75, 12.58	<0.001	94.0%	<0.001
Ou et al., 2024 [26]	11.34	7.87, 14.81	<0.001	97.0%	<0.001
Ragavan et al., 2023 [25]	11.23	7.62, 14.83	<0.001	97.0%	<0.001
Totaro et al., 2024 [22]	11.35	7.88, 14.81	<0.001	96.9%	<0.001
All studies	10.69	7.69, 13.69	<0.001	96.4%	<0.001
Estimated blood loss (ml)					
Studies	Effect size	95% CI	P-value	Heterogeneity	Het. P-value
Alfano et al., 2023 [3]	216.70	156.54, 276.85	<0.001	98.8%	<0.001
Antonelli et.al., 2024 [28]	216.11	155.86, 276.36	<0.001	98.8%	<0.001
Bravi et al., 2023 [23]	203.06	155.12, 251.01	<0.001	98.0%	<0.001
Brime Menendez et al., 2023 [18]	220.69	158.06, 283.32	<0.001	97.2%	<0.001
Jaffer et al., 2023 [21]	236.57	180.03, 293.10	<0.001	98.1%	<0.001
Ng et al., 2023 [20]	226.21	163.54, 288.88	<0.001	98.8%	<0.001

Olsen et al., 2023 [24]	217.24	157.20, 277.27	<0.001	98.8%	<0.001
Ou et al., 2024 [26]	227.06	165.21, 288.92	<0.001	98.9%	<0.001
Tedesco et al., 2023 [19]	230.98	170.25, 291.72	<0.001	98.8%	<0.001
Territo et al., 2023 [27]	226.13	163.61, 288.66	<0.001	98.9%	<0.001
Totaro et al., 2024 [22]	236.60	180.08, 293.11	<0.001	97.9%	<0.001
All studies	223.46	166.75, 280.17	<0.001	98.7%	<0.001
Length of stay (days)					
Studies	Effect size	95% CI	P-value	Heterogeneity	Het. P-value
Alfano et al., 2023 [3]	2.86	1.64, 4.09	<0.001	99.9%	<0.001
Bravi et al., 2023 [23]	2.75	1.51, 3.99	<0.001	100.0%	<0.001
Brime Menendez et al., 2024 [18]	2.84	1.60, 4.07	<0.001	100.0%	<0.001
Jaffer et al., 2023 [21]	2.92	1.72, 4.12	<0.001	100.0%	<0.001
Ng et al., 2023 [20]	2.75	1.51, 3.99	<0.001	100.0%	<0.001
Olsen et al., 2023 [24]	2.98	1.81, 4.14	<0.001	99.9%	<0.001
Ou et al., 2024 [26]	2.29	1.61, 2.97	<0.001	99.9%	<0.001
Ragavan et al., 2023 [25]	2.97	1.81, 4.13	<0.001	100.0%	<0.001
Territo et al., 2023 [27]	2.75	1.52, 3.98	<0.001	100.0%	<0.001

Totaro et al., 2024 [22]	2.64	1.43, 3.84	<0.001	100.0%	<0.001
All studies	2.78	1.67, 3.89	<0.001	100.0%	<0.001
Time to catheter removal (days)					
Studies	Effect size	95% CI	P-value	Heterogeneity	Het. P-value
Alfano et al., 2023 [3]	8.52	5.32, 11.71	<0.001	100.0%	<0.001
Bravi et al., 2023 [23]	9.23	6.78, 11.68	<0.001	99.9%	<0.001
Brime Menendez et al., 2023 [18]	7.85	4.81, 10.89	<0.001	100.0%	<0.001
Jaffer et al., 2023 [21]	8.04	5.02, 11.07	<0.001	100.0%	<0.001
Ng et al., 2023 [20]	8.52	5.32, 11.71	<0.001	100.0%	<0.001
Ou et al., 2024 [26]	8.51	5.32, 11.70	<0.001	100.0%	<0.001
Ragavan et al., 2023 [25]	8.52	5.32, 11.71	<0.001	100.0%	<0.001
Totaro et al., 2024 [22]	7.25	4.96, 9.54	<0.001	100.0%	<0.001
All studies	8.31	5.53, 11.09	<0.001	100.0%	<0.001
Positive surgical margins rate					
Studies	Effect size	95% CI	P-value	Heterogeneity	Het. P-value
Alfano et al., 2023 [3]	0.191	0.115, 0.278	<0.001	73.4%	<0.001

Bravi et al., 2023 [23]	0.219	0.139, 0.309	<0.001	61.3%	0.012
Brime Menendez et al., 2023 [18]	0.202	0.115, 0.304	<0.001	75.1%	<0.001
Jaffer et al., 2023 [21]	0.227	0.158, 0.303	<0.001	60.1%	0.014
Olsen et al., 2023 [24]	0.186	0.112, 0.271	<0.001	71.9%	0.001
Ou et al., 2024 [26]	0.201	0.119, 0.296	<0.001	75.1%	<0.001
Ragavan et al., 2023 [25]	0.198	0.119, 0.289	<0.001	74.8%	<0.001
Territo et al., 2023 [27]	0.193	0.116, 0.282	<0.001	74.1%	<0.001
Totaro et al., 2024 [22]	0.187	0.108, 0.279	<0.001	66.7%	0.004
All studies	0.200	0.126, 0.285	<0.001	71.5%	<0.001
Undetectable PSA rate at first follow-up after surgery					
Studies	Effect size	95% CI	P-value	Heterogeneity	Het. P-value
Alfano et al., 2023 [3]	0.922	0.847, 0.974	<0.001	60.8%	0.110
Bravi et al., 2023 [23]	0.946	0.922, 0.993	<0.001	0.0%	0.340
Totaro et al., 2024 [22]	0.945	0.789, 1.000	<0.001	63.3%	0.099
All studies	0.942	0.877, 0.986	<0.001	48.9%	0.141
Clavien-Dindo ≥2 complications rate					

Studies	Effect size	95% CI	P-value	Heterogeneity	Het. P-value
Alfano et al., 2023 [3]	0.040	0.008, 0.087	<0.001	66.6%	0.001
Bravi et al., 2023 [23]	0.041	0.006, 0.097	<0.001	65.7%	0.002
Brime Menendez et al., 2023 [18]	0.048	0.011, 0.100	<0.001	64.2%	0.003
Jaffer et al., 2023 [21]	0.026	0.005, 0.056	<0.001	42.8%	0.072
Ng et al., 2023 [20]	0.040	0.009, 0.085	<0.001	65.9%	0.002
Olsen et al., 2023 [24]	0.037	0.007, 0.082	<0.001	65.2%	0.002
Ou et al., 2024 [26]	0.048	0.013, 0.098	<0.001	64.4%	0.003
Ragavan et al., 2023 [25]	0.046	0.012, 0.095	<0.001	66.3%	0.002
Tedesco et al., 2023 [19]	0.048	0.013, 0.098	<0.001	64.4%	0.003
Territo et al., 2023 [27]	0.034	0.006, 0.075	<0.001	61.5%	0.005
Totaro et al., 2024 [22]	0.047	0.010, 0.103	<0.001	64.2%	0.003
All studies	0.041	0.010, 0.085	<0.001	63.6%	0.002
Social continence rate at 3 months					
Studies	Effect size	95% CI	P-value	Heterogeneity	Het. P-value
Bravi et al., 2023 [23]	0.821	0.707, 0.913	<0.001	72.3%	0.003
Brime Menendez et al., 2023 [18]	0.803	0.708, 0.884	<0.001	65.8%	0.012

Ng et al., 2023 [20]	0.819	0.732, 0.893	<0.001	72.3%	0.003
Olsen et al., 2023 [24]	0.839	0.766, 0.902	<0.001	61.6%	0.023
Ou et al., 2024 [26]	0.827	0.735, 0.904	<0.001	71.8%	0.003
Ragavan et al., 2023 [25]	0.795	0.728, 0.854	<0.001	48.4%	0.084
Totaro et al., 2024 [22]	0.833	0.732, 0.916	<0.001	68.0%	0.008
All studies	0.819	0.738, 0.889	<0.001	66.9%	0.006

Supplementary Table S9. *Comparison of technical insights between Hugo^{TM} RAS and daVinci systems*

	**daVinci Surgical System**	**HUGO^{TM} RAS System**
Console [41,42]	Closed console<br>Surgeon most deeply "immersed" during the procedure	Open console<br>Improved surgeon awareness of the theatre<br>Multitasking

	Less susceptibility to external distractions<br>Microphone system for communication<br>Needs to withdraw their focus to visually connect with the operating room.	Face-to-face communication – easier team communication (facilitating surgical training) [22,23,25,27]<br>External factors can disturb the surgeon's concentration
Seated position [41,42]	Constrained postures – no possibility to vary the working posture<br>The surgeon has to lean forward slightly to have a great view of the screen.	Move the head freely to adopt various working postures.<br>Better ergonomic position because it is possible to sit more comfortably.
Arm cart/system tower	<u>Individual arm cart</u><br>External range of motion is potentially facilitated without arm collisions [41]<br>Design of setup joints with limited freedom of movement is such so as to facilitate port placement [41]<br>Less spatial constraint in operating room [41]<br>Assistant surgeon and scrub nurse comfort is enhanced due to "spacious" table side by daVinci's smaller arms [28]<br>There are fewer cables on the operating room floor [41]	<u>Modular arm cart</u><br>Facilitates multi-quadrant surgery [27]<br>Provides a greater range of angle for docking<br>More challenging and time-consuming docking process [3,22,23,28]<br>More spatial constraint in operating room [25,26,27]<br>Assistant surgeon and scrub nurse discomfort is due to the "crowded" table side by HUGO RAS's larger arms [28]<br>There are more cables on the operating room floor [41]
	3D HD vision system [44]	3D HD vision system [43]

Vision system	Stereoscopic technology – more realistic display [26]	Passive 3D display lag in realism [26]<br>Camera movement automatically follows the 3D glasses movement of the surgeon, facilitating a natural shift in task focus
Manual control	Pincer-grip master controller [44]<br>Endowrist instruments:<br>7° of freedom<br>90° of articulation<br>Intuitive motion and finger-tip control<br>Motion scaling and tremor filtration [44]	Pistol-grip controller [43]<br>Scaling factor for wrist rotation up to 2x and rotation range up to 520°[23]<br>Adjustable scaling for wrist rotation facilitates suture in deep cavities [27]<br>Tremor filtration [43]
Foot pedal array [41, 42]	Swap pedal instrument: one click is enough and the arm in use changes<br>The right foot controls the energy instruments, while the left foot activates the clutch and endoscope<br>A second optional console allows for tandem surgery and training	Swap pedal instrument.: it is necessary to keep it pressed for 1.5 seconds for the change occur<br>Monopolar and bipolar power activation: initially, press the pedal for 1.5 seconds, release it, and then activate it again<br>LigaSure pedals to the left of the bipolar pedal (given that 90% of surgeons are right-handed, it would be more intuitive for the LigaSure pedals to be positioned to the right of the bipolar pedal)
	Quick-release levers speed expedite instrument changes during surgery [44]	Disposable instruments [43]<br>Advanced energy, stapling devices, and clip appliers not available [41,42]

Instruments	Force bipolar: DualGrip technology allows for adjustable grip strength (default or strong) via the foot pedal [44]<br><br>EndoWrist technology provides full wristed dexterity [44]<br><br>daVinci energy enables rapid sealing while maintaining low temperatures [44]	Lack of a robotic instrument for "gentle" grasping and traction [22]<br><br>Scissors may jump slightly upon closure, especially when used with active monopolar diathermy in a "cut-burn" technique [42]<br><br>Higher incidence of malfunctioning events (20 out of 50 procedures). Issues include platform battery supply alarm, system power failure, arm conflicts, scissors rupture, malfunctioning Maryland bipolar forceps, and failed fourth arm calibration [28]
Artificial Intelligence Tool	My Intuitive software [44]	Touch Surgery™ software: identifies and automatically segments steps of the procedure (streamlining teaching) [43]

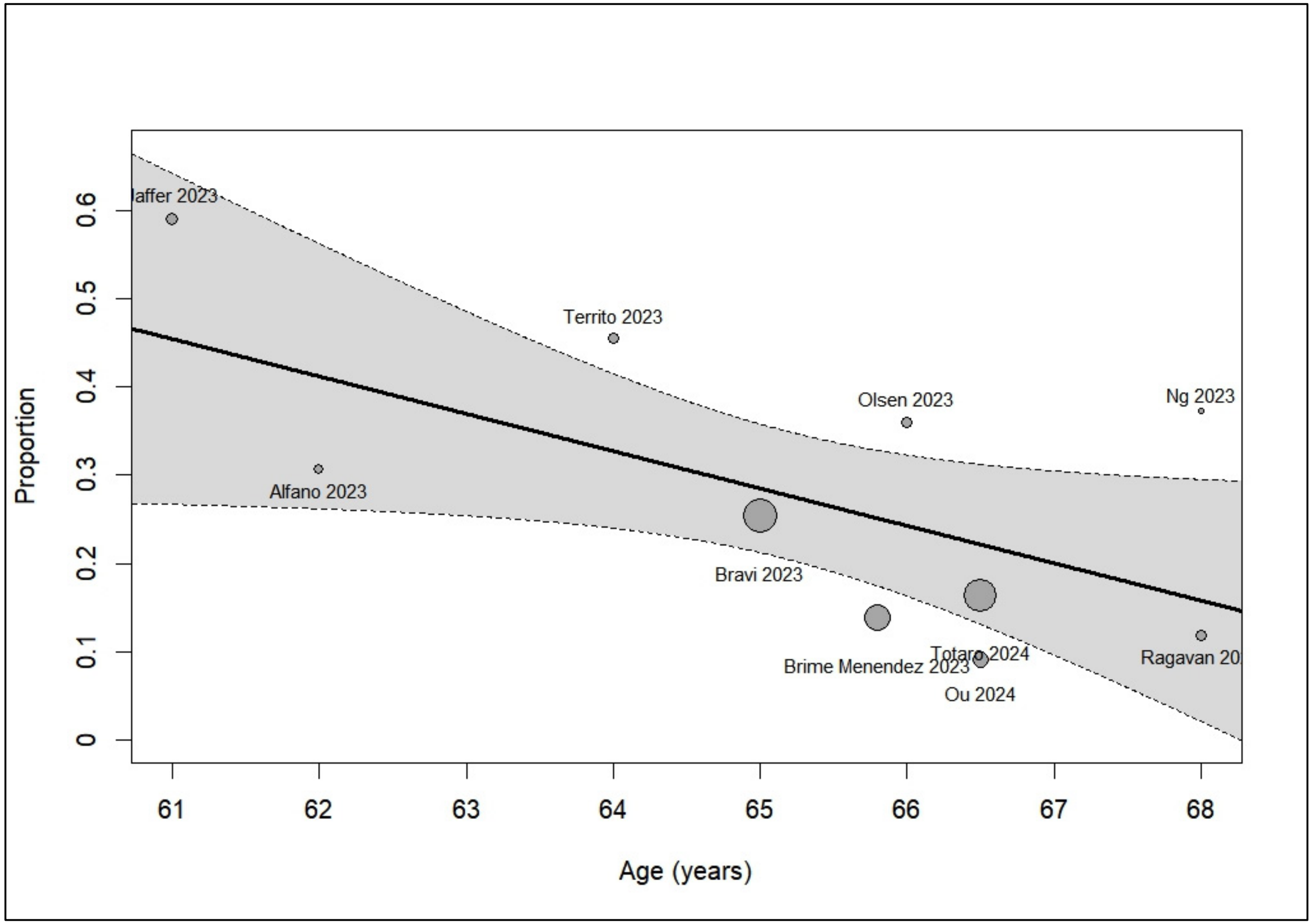

Supplementary Figure S1. *Bubble plot of Clavien-Dindo ≥2 complications*
